# Inhaled corticosteroids for the treatment of COVID-19

**DOI:** 10.1183/16000617.0099-2022

**Published:** 2022-11-30

**Authors:** Mona Bafadhel, Rosa Faner, Camille Taillé, Richard E.K. Russell, Tobias Welte, Peter J. Barnes, Alvar Agustí

**Affiliations:** 1 King’s Centre for Lung Health, School of Immunology and Microbial Sciences, Faculty of Life Sciences and Medicine, King’s College London, London, UK; 2 CIBER Enfermedades Respiratorias, IDIBAPS, Barcelona, Spain; 3 Department of Pulmonary Diseases, University Hospital Bichat-Claude Bernard, AP-HP Nord, University of Paris, Paris, France; 4 Department of Pulmonary and Infectious Diseases, Hannover University School of Medicine, Hannover, Germany; 5 National Heart and Lung Institute, Imperial College London, London, UK; 6 Cátedra de Salud Respiratoria (University of Barcelona), Respiratory Institute (Hospital Clinic Barcelona), IDIBAPS and CIBERES, Barcelona, Spain

## Abstract

The severe acute respiratory syndrome coronavirus 2 (SARS-CoV-2) pandemic has caused severe illness and mortality for millions worldwide. Despite the development, approval and rollout of vaccination programmes globally to prevent infection by SARS-CoV-2 and the development of coronavirus disease 2019 (COVID-19), treatments are still urgently needed to improve outcomes. Early in the pandemic it was observed that patients with pre-existing asthma or COPD were underrepresented among those with COVID-19. Evidence from clinical studies indicates that the inhaled corticosteroids (ICS) routinely taken for asthma and COPD could have had a protective role in preventing severe COVID-19 and, therefore, may be a promising treatment for COVID-19. This review summarises the evidence supporting the beneficial effects of ICS on outcomes in patients with COVID-19 and explores the potential protective mechanisms.

## Abstract 2

**Treatments are urgently needed to improve outcomes for patients with coronavirus disease 2019 (COVID-19) infection. This review summarises the evidence for the potential protective role of inhaled corticosteroids in preventing severe COVID-19** https://bit.ly/3yCAvfn

## Introduction

The pandemic of the severe acute respiratory syndrome coronavirus 2 (SARS-CoV-2), causing coronavirus disease 2019 (COVID-19), has placed an unprecedented burden on healthcare systems globally. The first reported human case of COVID-19 was in December 2019, and the outbreak has resulted in over 600 million cases of the disease and over 6 million deaths worldwide to 15 September 2022 [1, 2]. COVID-19 is a respiratory infection that in mild cases causes fever, persistent cough and shortness of breath, but in severe cases can cause serious lung damage (requiring hospitalisation and often critical care) and death. Currently, systemic corticosteroids alongside the Janus kinase inhibitor baricitinib or an interleukin (IL)-6 receptor blocker are the only drugs strongly recommended by the World Health Organization for the treatment of severe COVID-19 [3]. Mass vaccination programmes using a variety of different COVID-19 vaccines have by now administered >12 billion doses globally [4], but there is still an urgent need for treatments to halt disease progression and enhance recovery from COVID-19.

Viral upper respiratory tract infections commonly trigger exacerbations of asthma and COPD, with the majority of exacerbations linked to the common cold caused by rhinoviruses and coronaviruses, as well as influenza and respiratory syncytial virus [5–8]. Based on existing knowledge of viral respiratory diseases, it was expected that patients with pre-existing chronic lung conditions would be overrepresented among patients hospitalised with COVID-19 and associated with worse outcomes, but this has not transpired [9, 10]. Several, nonmutually exclusive reasons have been suggested that may explain these observations. First, it is possible that respiratory diseases were underdiagnosed or underreported in different countries during the early COVID-19 pandemic, as highlighted in reports from China [11, 12]; second, the dominant airway inflammation associated with the patients’ underlying disease might have provided some protection from infection [13, 14]; third, perhaps patients with chronic lung disease behaved more cautiously than the general population early in the COVID-19 pandemic [13, 15], although a survey in France reported that patients with asthma did not behave differently from patients with other chronic diseases [16]; or fourth, treatments used in asthma and COPD, such as inhaled corticosteroids (ICS) may have had a protective effect [11, 13, 15]. In fact, several clinical studies and meta-analyses have suggested that the use of ICS in the treatment of patients with mild or moderate COVID-19 may prevent disease progression [17–22] and may also reduce the risk of disease progression in patients hospitalised due to COVID-19 pneumonia [23].

In this review we summarise the biological, epidemiological and clinical evidence available to date in relation to COVID-19 in patients with asthma or COPD, discuss the association between ICS use and better COVID-19 outcomes, and propose a case for adding ICS to the current armamentarium of treatments for use in COVID-19.

## Search strategy

Data for this narrative review were identified by searches of PubMed (https://pubmed.ncbi.nlm.nih.gov), and references from relevant articles, using the following search terms: COVID-19 AND Inhaled Corticosteroid; Coronavirus AND Inhaled Corticosteroid; COVID-19 AND COPD OR Chronic Obstructive Pulmonary Disease; COVID-19 AND Asthma; Coronavirus AND COPD OR Chronic Obstructive Pulmonary Disease; Coronavirus AND Asthma; Inhaled Corticosteroids AND Virus; COVID-19 AND Asthma OR COPD AND Prevalence. Only articles published in English or preprint between January 2020 and March 2022 were included. Additional search terms (non-COVID-19 searches) were Virus AND Exacerbations AND Budesonide/Ciclesonide/Beclomethasone/Mometasone/Flunisolide/Fluticasone; Virus AND Budesonide/Ciclesonide/Beclomethasone/Mometasone/Flunisolide/Fluticasone. Only articles published in English between January 2000 and January 2021 were included.

## Basic SARS-CoV-2 biology

**FIGURE 1:**
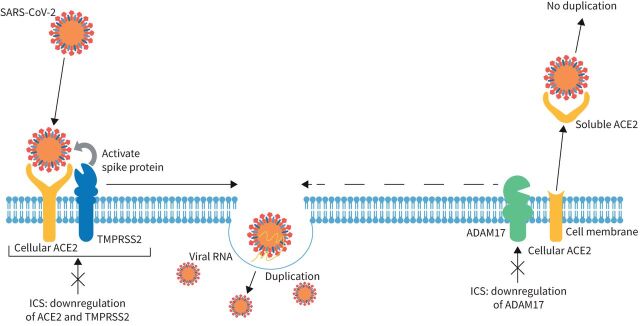
Mechanisms of severe acute respiratory syndrome coronavirus 2 (SARS-CoV-2) entry into cells and potential effects of inhaled corticosteroids (ICS). SARS-CoV-2 binding to membrane-bound angiotensin-converting enzyme 2 (ACE2) leads to type II transmembrane serine protease (TMPRSS2) priming the SARS-CoV-2 spike protein, resulting in its cleavage. This enables the virus to enter the cell *via* fusion with the cell membrane. Disintegrin and metalloproteinase 17 (ADAM17) can cleave the ectodomain of ACE2, resulting in soluble ACE2, which binds SARS-CoV-2, but does not lead to the virus infecting the cell. The role of ADAM17 in infection is not clear, but it may contribute to fusion of SARS-CoV-2 virus particles with the cell membrane (represented by dashed line arrow). ICS may modify severity of coronavirus disease 2019 infection by downregulating components important for virus attachment and cell entry, such as ACE2 [85, 87, 88], TMPRSS2 [85] and ADAM17 [89]. Reproduced from [25].

SARS-CoV-2 is a single-stranded RNA virus that uses its spike (S) proteins, composed of two subunits (S1 and S2) [24], to enter host cells *via* angiotensin-converting enzyme 2 (ACE2) [25]. The proposed mechanisms by which SARS-CoV-2 infects cells is shown in figure 1 [25]. ACE2 is expressed in the upper respiratory tract, including the nasal epithelium and the endothelial cells of several organs, and it is also abundantly expressed on alveolar epithelial cells in the lungs [26]. The interactions of ACE2 with the type II transmembrane serine protease (TMPRSS2) and a disintegrin and metalloproteinase 17 (ADAM17) determine virus entry into cells [24, 27].

Lung damage is a result of the cytopathic effects on infected pneumocytes [28, 29], as well as the virus-induced immune response [30], and can include alveolar damage, hyaline membrane formation and pulmonary oedema [29]. In severe cases, SARS-CoV-2 infection is characterised by high serum levels of pro-inflammatory cytokines and chemokines, coupled with a delayed or inadequate interferon response [1]. Infection can cause severe pneumonia leading to acute respiratory distress syndrome (ARDS) [29], resulting in complications that require supportive mechanical ventilation [31]. SARS-CoV-2 can also have systemic effects beyond the lungs, resulting in damage to other organs [32].

## Epidemiology of COVID-19 in patients with asthma and COPD

A summary of real-world studies investigating the association of asthma and COPD with COVID-19 is presented in table 1 [12, 33–43]. Studies in China, the USA and Ireland, as well as some systematic reviews, have demonstrated that there was either a lower prevalence of asthma among patients with COVID-19, compared with the general population [35, 37], or a prevalence comparable to the general population [40, 41]. In a study in Russia, the prevalence of COPD among patients with COVID-19 was comparable to the general population [33]. Furthermore, in a study conducted in China, the prevalence of asthma and COPD was lower in patients with COVID-19 in comparison to some other comorbidities, such as hypertension and diabetes [12].

**TABLE 1:** Studies investigating the association of asthma and COPD in coronavirus disease 2019 (COVID-19) patients

**Study**	**Study design**	**Location**	**Cohort**	**Setting**	**Results**
**Guan *et al.* [12]**	Observational	China	COVID-19 positive; n=1590	Hospital	Lower prevalence of COPD (1.5%) and asthma (0%) *versus* other comorbidities – hypertension (16.9%) and diabetes (8.2%)
**Avdeev *et al.* [33]**	Observational	Russia	COVID-19 (positive or pending with severe acute respiratory infection and typical CT); n=1307	ICU	Low prevalence of bronchial asthma (1.8%) and COPD (3.1%), not higher than in the general population
**Robinson *et al.* [34]**	Observational	USA	COVID-19 positive; n=866	Hospital	Hazard ratio 0.52 (95% CI 0.30–0.90) for ICU admission and 0.42 (95% CI 0.21–0.81) for mechanical ventilation for patients with asthma *versus* patients without asthma
**Ho *et al.* [35]**	Observational	USA	COVID-19 positive; n=10 523	Hospital inpatient and outpatient	Odds ratio 0.43 (95% CI 0.28–0.64) for hospitalisation, 0.51 (95% CI 0.41–0.64) for ICU admission and 0.64 (95% CI 0.53–0.77) for mortality for patients with asthma *versus* patients without asthma
**Cao *et al.* [36]**	Observational	USA	COVID-19 positive; n=343	Medical centre	Odds ratio 1.00 (95% CI 0.34–3.28) for hospitalisation, 0.59 (95% CI 0.31–1.08) for ICU admission, 1.10 (95% CI 0.56–2.12) for mechanical ventilation, 0.73 (95% CI 0.30–1.64) for in-hospital mortality due to COVID-19 for patients with asthma *versus* patients without asthma
**Li *et al.* [37]**	Observational	China	COVID-19 positive; n=548	Hospital	Low proportion of patients with COVID-19 who had asthma (0.9%)
**Izquierdo *et al.* [38]**	Observational	Spain	Asthma; n=71 182	EHRs: primary care, hospital inpatient, outpatient, emergency department	Odds ratio 2.29 (95% CI 4.35–6.66) for mortality for patients with asthma and COVID-19 *versus* patients with asthma without COVID-19
**Choi *et al.* [39]**	Observational	South Korea	COVID-19 positive; n=7590	Medical claims (including hospital inpatient and outpatient)	Patients with step 5 (more severe) asthma had a more prolonged stay in hospital *versus* patients with step 1 asthma
**Butler *et al.* [40]**	Observational	Ireland	COVID-19 positive; n=193	Hospital	Prevalence of asthma (8.8%) *versus* general population (7.0%)
**Sunjaya *et al.* [41]**	Systematic review	Multiple countries	COVID-19 positive; n=349 592 overall, n=369 405 for risk ratio reduction analysis	Primary care, hospital, mixed	Prevalence of asthma was 7.46% (95% CI 6.25–8.67). Showed a 14% (95% CI 0.80–0.94) and 13% (95% CI 0.77–0.99) risk ratio reduction for acquiring and being hospitalised for COVID-19, respectively, for patients with asthma *versus* no asthma
**Rogliani *et al.* [42]**	Systematic review	Multiple countries	COVID-19 positive; n=8476	Hospital	Relative risk of hospitalisation reduced in patients with asthma (0.86, 95% CI 0.77–0.97) and COPD (0.46, 95% CI 0.40–0.52) *versus* general population
**Liu *et al.* [43]**	Systematic review	Multiple countries	COVID-19; n=410 382 overall, n=5000 for risk ratio analysis	Nonhospital, hospital, ICU	Risk ratio 0.65 (95% CI 0.43–0.98) for mortality for patients with asthma *versus* patients without asthma

CT: computed tomography; EHR: electronic health record; ICU: intensive care unit.

Overall, the available evidence indicates that patients with asthma [34–36, 41–44] did not have an increased risk, or had a lower risk, of severe disease, hospitalisation or mortality when infected with SARS-CoV-2, compared with patients without asthma. However, the association is less clear for patients with COPD [42, 45], with a pooled analysis of epidemiological studies showing a significantly reduced relative risk of hospitalisation due to COVID-19 [42] and a systematic review and meta-analysis suggesting that patients with pre-existing COPD have greater than three times the risk of mortality and severe COVID-19 [45]. Other studies also present an inconsistent picture of the risk of worse COVID-19 outcomes with chronic respiratory diseases, although these could be partly explained by differences in the populations studied. For example, in a study in France of hospitalised patients with COVID-19, those with a chronic respiratory disease had an increased risk of severe disease and mortality compared with patients without a chronic respiratory disease [10]. However, in patients with chronic respiratory diseases, the risk of severe COVID-19 was lower than the risk of severe influenza observed in other studies [10]. A study in Spain reported an increase in mortality among patients with asthma and COVID-19, although the comparator population in this retrospective investigation was limited to patients with asthma [38]. Additionally, of note is that in 2020 and early 2021, several studies have observed that the most frequently reported comorbidities associated with hospitalisation and worse outcomes in COVID-19 included hypertension, obesity, diabetes and dyslipidaemia [11, 12, 38, 46]. However, one case series from China reported that chronic respiratory diseases had the third highest case fatality rate after cardiovascular disease and diabetes [47].

## Asthma/COPD biology and COVID-19

Airway inflammation associated with patients’ underlying chronic respiratory diseases could potentially provide some protection against severe COVID-19. For instance, there may be cross-regulation between the type 2 (T2) immune response in allergic asthma and the interferon-mediated immune response in COVID-19 [14]. Allergic asthma is characterised by the presence of T2 cytokines, some of which can inhibit the production of pro-inflammatory cytokines, and increased numbers of eosinophils (a T2 marker) [48]. Asthma and atopy are also associated with reduced expression of ACE2, which is needed for SARS-CoV-2 to infect cells [49]. A retrospective study of 951 patients with asthma and COVID-19 found that blood eosinophil levels ≥150 cells·µL^−1^ were associated with a lower likelihood of being admitted to hospital compared with eosinophil levels <150 cells·µL^−1^, and the mortality rate was lower for hospitalised patients whose eosinophil levels increased to ≥150 cells·µL^−1^ [50]. Another retrospective cohort study of more than 10 000 patients with COVID-19 found that patients with and without asthma and blood eosinophils ≥200 cells·µL^−1^ had a significantly lower risk of mortality than those with lower eosinophil levels, although it was not reported how many of these patients were on ICS treatment [35, 51]. A registry study including >46 000 patients with COVID-19, however, has demonstrated an association between eosinophil levels and risk for COVID-19 outcomes that varies by ICS treatment. The authors showed that the predicted probability for COVID-19-related hospitalisation, intensive care unit admission or in-hospital mortality was higher among people with eosinophil levels ≥150 cells·µL^−1^ who were not treated with ICS, but not among those who were [52]. In contrast, studies have found that a greater severity of airflow limitation in patients with COPD (regardless of smoking status) is related to higher expression of ACE2, which could increase the susceptibility of these patients to SARS-CoV-2 infection [53, 54].

## Antiviral effects of ICS

### Common cold viruses

ICS are frequently used in the treatment of patients with asthma and COPD in combination with bronchodilators [55, 56]. Multiple studies have shown that ICS, in combination with long-acting β_2_-agonists, is an effective treatment for reducing virus-induced asthma exacerbations [57, 58]. Evidence for the importance of ICS in this combination supports its potential to alleviate the effects of SARS-CoV-2 infection in the lungs [59, 60]. For instance, ICS use has previously been found to be protective against hospitalisation for rhinovirus-induced asthma exacerbations [60]. Furthermore, a retrospective study of more than 12 000 patients with asthma, which investigated different regimens of asthma treatments on reducing common cold-related severe exacerbations, found that the dosage and/or timing of ICS administration was an important factor influencing the risk of cold-related exacerbations [59].

*In vitro* studies suggest mechanisms for how ICS (and β_2_-agonists) might act on cells of the lungs to reduce exacerbations caused by common cold viruses. Budesonide and formoterol have been shown to reduce type 14 rhinovirus-induced pro-inflammatory cytokine release and inhibit viral RNA replication in human tracheal epithelial cells [61]. A budesonide/glycopyrronium/formoterol combination inhibited human coronavirus-induced pro-inflammatory cytokine production and virus replication in primary human nasal and tracheal epithelial cells [62]. Another study showed that budesonide and formoterol, as well as budesonide alone, inhibited rhinovirus-induced chemokines in primary normal human bronchial epithelial (NHBE) cells [63]. Additionally, fluticasone propionate and budesonide have both been shown to inhibit viral mimic poly-(I:C)-induced chemokine release in the cell line 16HBE and human primary bronchial epithelial cells, fluticasone propionate inhibited respiratory syncytial virus-induced chemokine release in the cell line BEAS-2B and fluticasone propionate alone or with salmeterol inhibited rhinovirus-induced cytokine release, also from BEAS-2B cells and from NHBE cells [64–66].

## ICS use in patients with COVID-19

Potential protective effects of ICS on COVID-19 outcomes in patients with asthma or COPD have been observed. In a retrospective study of 71 182 patients with asthma, in Spain, fewer patients who were hospitalised for COVID-19 were ICS users, *versus* nonhospitalised patients [38]. A retrospective study in South Korea found that the use of ICS by patients with asthma tended to be associated with a reduced risk of COVID-19, although this was not statistically significant [67]. Another retrospective study, in Israel, showed that ICS use in patients with mild bronchial asthma did not have any impact on whether they tested positive for COVID-19 [68]. A retrospective study in Denmark found that there were no positive or negative effects of existing ICS use on intensive care admission or mortality, in patients hospitalised with SARS-CoV-2 infection, although a sensitivity analysis suggested that budesonide use was associated with a reduced risk of severe COVID-19 outcomes relative to use of fluticasone, compared with no ICS use or use of bronchodilators [69]. A retrospective study in the UK showed that among over 65 000 patients ≥50 years of age who were hospitalised for COVID-19, there was a reduced mortality risk for those with asthma (but not for those with COPD) who were using ICS within 2 weeks of admission [70]. However, a limitation of this study was that asthma severity status was based solely on medication history. Conversely, in the large observational OpenSAFELY study in the UK, the risk of COVID-19-related death was reported to be higher in patients with asthma or COPD who were using ICS compared with those using other noncorticosteroid respiratory medications, albeit again this study was not able to account for differences in underlying disease severity [71, 72]. Additionally, a retrospective chart review of 787 patients with asthma hospitalised for symptomatic COVID-19 showed that patients already on ICS were 1.6 times more likely to be discharged on oxygen than patients not on ICS, though whether this reflected underlying asthma severity or use of ICS is not clear [73]. Another retrospective, observational study including 275 hospitalised COVID-19 patients with asthma reported that the number of days on ventilation was significantly increased in patients already receiving ICS therapy *versus* those who were not; however, being on ICS therapy at admission did not alter overall COVID-19 severity, disease complications or mortality [74]. These studies and their findings highlight the difficulties in accounting for the heterogeneity in underlying respiratory disease and disease severity when investigating the benefits of ICS in COVID-19 in the real world. Furthermore, the use of oral rescue packs (*e.g.* oral corticosteroids) by patients with asthma or COPD who develop COVID-19 symptoms is also a consideration in the interpretation of the real-world data for any benefit of ICS, although this does not impact results of some of the randomised, clinical trials as the authors specify recent inhaled or systemic glucocorticoids as an exclusion criterion [17, 18, 23].

The first randomised clinical trial to examine the effect of ICS in patients with COVID-19 was the phase II Steroids in COVID-19 (STOIC) proof-of-concept study, which examined the effect of inhaled budesonide in early COVID-19 in adults aged 18 and over [17]. Using daily home monitoring, the study showed that inhaled budesonide (800 µg twice daily) within 7 days of onset of mild COVID-19 symptoms reduced the likelihood of needing urgent care (including emergency department assessment or hospitalisation) by 91% (relative risk reduction) and reduced self-reported recovery time by 1 day, compared with usual care. The median time to resolution of symptoms (measured by the Influenza Patient-Reported Outcome questionnaire), was numerically improved by 1 day with budesonide treatment *versus* usual care. Additionally, a significantly greater proportion of patients was symptom free after 14 days with budesonide treatment. There was no change in viral load at the end of the 28-day treatment period. The Platform Randomised trial of INterventions against COVID-19 In older peoPLE (PRINCIPLE) study was a phase III clinical platform study in the UK [18]. The PRINCIPLE patient population included individuals ≥65 years old (or ≥50 years with underlying health conditions) who were randomised to receive inhaled budesonide (800 µg twice daily) within 14 days of COVID-19 symptoms or a positive test, or standard care. The results showed that recovery time was on average 3 days shorter and hospital admissions or deaths were 2% lower in patients receiving inhaled budesonide compared with patients receiving standard care, although this result did not meet the prespecified superiority threshold. Secondary outcomes in PRINCIPLE showed evidence of reduced time to sustained alleviation of all symptoms, as well as shortened time to initial reduction of symptom severity with budesonide treatment *versus* usual care. The phase IV investigator-initiated, open-label TACTIC trial recruited adults aged 18−80 in Argentina and Spain, hospitalised with COVID-19 pneumonia [23]. The study investigated the effects of the addition of inhaled budesonide to standard care, compared with standard care alone, on disease progression (a composite variable including the initiation of treatment with high-flow oxygen therapy, noninvasive or invasive ventilation, and/or death) at day 15 after starting treatment. Although the study was terminated early and lacked statistical power, the results suggested that inhaled budesonide (400 µg twice daily) in addition to standard care has a favourable safety profile and may reduce the risk of disease progression [23]. A randomised, open-label phase II trial to evaluate the efficacy of ciclesonide treatment for 14 days *versus* standard care showed that inhaled ciclesonide shortened viral shedding duration and may inhibit progression to acute respiratory failure in patients with mild-to-moderate COVID-19 [22]. However, an open-label phase III study to assess the efficacy of inhaled ciclesonide in reducing the risk of adverse outcomes in COVID-19 patients at risk of developing severe illness demonstrated insufficient evidence that ICS are beneficial for people with COVID-19 [75]. A phase III study in the USA that investigated the safety and efficacy of inhaled ciclesonide on nonhospitalised patients aged 12 and over with symptomatic COVID-19 showed that there was no difference in the time to alleviation of COVID-19-related symptoms with inhaled ciclesonide (320 µg twice daily for 30 days) compared with placebo [19]. However, patients who received ciclesonide had fewer emergency department visits or hospital admissions for reasons related to COVID-19 [19].

In May 2021, the European Medicines Agency COVID-19 Taskforce reviewed evidence for the treatment of outpatients with COVID-19 [17–19] and concluded that it was insufficient to show a benefit of ICS in people with COVID-19 [76]. A meta-analysis including data from STOIC [17], PRINCIPLE [18], the phase III US study [19] and CONTAIN, a Canadian, placebo-controlled trial of inhaled ciclesonide in adult outpatients with COVID-19 [77], compared the outcome of hospitalisation and showed a promising effect of ICS in preventing hospitalisation [20]. It was noted, however, that STOIC included a composite outcome of urgent care visits and hospitalisation; therefore, the effect on urgent care visits may have exaggerated the estimated effect on hospitalisations. Furthermore, potential bias was highlighted of unblinded providers being more likely to refer to urgent care when a patient was not on treatment [20]. A Cochrane review of ICS for the treatment of COVID-19 found moderate-certainty evidence that ICS *versus* standard of care in people with asymptomatic/mild COVID-19 probably reduces admission to hospital or death, and increases the resolution of all initial symptoms at day 14 [21]. The authors noted that the evidence identified came from studies in high-income settings – STOIC, PRINCIPLE and the phase III US study – prior to vaccination rollout and emergence of newer variants (delta, omicron) of SARS-Cov-2, yet assume no relevant effect on expected benefits and harms of ICS [21]. Details of ongoing, or recently completed but currently unpublished (as of March 2022), prospective randomised clinical trials investigating ICS therapy in SARS-CoV-2 infection are shown in table 2 [78–84].

**TABLE 2:** Ongoing clinical studies investigating inhaled corticosteroid treatment for coronavirus disease 2019 (COVID-19) in adults

**Trial no.**	**Name**	**Treatment**	**Setting**	**Primary outcome**	**n (planned)**	**Start date**	**Estimated or actual primary completion date**	**Location**
**JPRN-jRCTs031190269 [83]**	RACCO	Ciclesonide 400 µg three times daily for 7 days	Hospital	Incidence of pneumonia on day 8 following treatment	90	April 2020	October 2020	Japan
**NCT04381364 [84]**	HALT COVID-19	Ciclesonide 320 µg twice daily for 14 days *versus* standard care	Hospital	Duration (days) of received supplemental oxygen therapy within 30 days after study inclusion	446	May 2020	August 2021	Sweden
**EudraCT 2020-002208-37 [80]**	CIMMCov	Ciclesonide 320 µg twice daily for 28 days *versus* placebo	Primary care	Reduction in healthcare resource utilisation (renewed contact with GP, emergency department and/or admission to hospital) due to COVID-19-related symptoms	138	June 2020^#^	Ongoing^¶^	Denmark
**CTRI/2020/10/028581 [78]**		Budesonide Rotacaps 200 µg twice daily for 10–14 days in addition to standard care *versus* standard care alone	Primary care	Hospitalisation	1000	October 2020	December 2020	India
**CTRI/2021/05/033817 [79]**		Budesonide 400 µg (two puffs twice daily) for up to 28 days *versus* standard care alone	Primary care	Hospitalisation or emergency department attendance related to COVID-19	820	May 2021^+^	Unknown	India
**NCT04937543 [81]**	TRIVID	Beclomethasone 250 µg or beclomethasone/formoterol/glycopyrronium 100/6/12.5 µg (two doses twice daily) for 28 days in addition to standard care *versus* standard care alone	Primary care	Use of healthcare resources 28 days after treatment	260	June 2021	October 2021	Brazil
**NCT05054322 [82]**	FLOT	Fluticasone propionate 125 µg with spacer (four puffs twice daily) for 14 days in addition to standard care	Primary care	Incidence of adverse outcomes (oxygen therapy, systemic corticosteroids, hospitalisation, mechanical ventilation and mortality) in symptomatic patients at 28 days after study inclusion	500	September 2021	January 2022	Vietnam

^#^: Trial ongoing as of 11 January 2022 (date accessed). ^¶^: Date on which this record was first entered in the EudraCT database. ^+^: Date of first enrolment (India). GP: general practitioner.

There are several different, nonmutually exclusive mechanisms by which ICS could potentially modify the severity of COVID-19 infection. Firstly, they may prevent SARS-CoV-2 infection by downregulating components important for virus attachment and cell entry (figure 1), as ICS use has been associated with lower expression of ACE2 and TMPRSS2 [85]. The ACE2 gene can be stimulated by interferon [86], and a study reported that ICS downregulated ACE2 expression, through suppression of type I interferon in airway epithelial cells from patients with COPD [87]. In addition, fluticasone propionate, an ICS that is also used intranasally, has been shown to suppress viral mimic-induced ACE2 gene and protein expression in primary human nasal epithelial cells [88]. Downregulation of ADAM17 gene expression has also been reported in association with ICS, although the implication for SARS-CoV-2 infection is unclear [89]. Secondly, some ICS have shown antiviral activity against SARS-CoV-2 *in vitro*. One *in vitro* study demonstrated that the ICS ciclesonide had antiviral activity against SARS-CoV-2 [90] and in another study, some but not all ICS therapies blocked SARS-CoV-2 replication *in vitro*, a finding that warrants further investigation [91]. Another study found that budesonide had an antiviral effect against SARS-CoV-2 wild-type strain B1.1.70 and two variants, B.1.1.7 (alpha) and B1.351 (beta), *in vitro* [92]. Thirdly, in asthma, ICS reduce airway inflammation by suppressing gene transcription of inflammatory cytokines and by inhibiting recruitment of inflammatory cells [93], so they can potentially prevent the hyperinflammation seen in SARS-CoV-2 infection, as mechanistic modelling has indicated [94]. A study assessing inflammatory mediators in the nasal mucosa and blood of patients from the STOIC clinical trial and a SARS-CoV-2-negative cohort provided a mechanistic insight into the effects of inhaled budesonide on early COVID-19 infection and how this could lead to shortened recovery time in patients treated with budesonide [95]. It was found that there was a distinctive nasal inflammatory response in early COVID-19, comprising a combined type I and type II interferon response and a type 1 (T1) and T2 cytokine- and chemokine-mediated response [95, 96]. Early administration of budesonide was found to attenuate inflammation by significantly suppressing IL-33 and interferon-γ, suggesting a dampening of the interferon response and reduced epithelial damage. Furthermore, the chemokine CCL17 was significantly elevated, suggesting an improved T-cell response [95]. Finally, a study showed that ICS given to patients at risk of developing ARDS improved blood oxygenation and reduced this risk, although inhaled β-agonists may have also played a role in this setting [97].

## Clinical practice considerations

The availability of effective SARS-CoV-2 vaccines was a key step forward in our fight against the pandemic, but we still need effective therapies for individuals who develop COVID-19. The lungs are the main site of the effects of SARS-CoV-2 infection and the use of ICS in respiratory disease to directly target the lungs is well established. Available evidence reviewed here indicates that the use of ICS – specifically budesonide or ciclesonide – can improve outcomes of COVID-19. However, on the basis of evidence from the STOIC [17] and PRINCIPLE [18] trials, guidance has been issued in the UK that ICS (specifically budesonide) are only recommended to treat COVID-19 as part of a clinical trial, although should not be discontinued if a patient is already on treatment. Although the benefit of inhaled budesonide in reducing time to recovery from COVID-19 was acknowledged, evidence was considered limited as STOIC was terminated early and did not meet the target sample size. Furthermore, as study populations included primarily older people, the results are not generalisable to other age groups [98]. However, inhaled budesonide or ciclesonide is recommended as a treatment to consider for mild COVID-19 in Canada (British Columbia), and inhaled budesonide in India, Russia and Saudi Arabia [99–102].

The worldwide availability and affordability of ICS (budesonide is on the World Health Organization's list of essential medicines [103]), including in generic form, facilitates their use where there are regional disparities in the management of the COVID-19 pandemic. This is especially important in regions with less advanced vaccination programmes, less developed healthcare resources and where there is concern with SARS-CoV-2 variants and reduced vaccine efficacy.

There are indications that more severe and symptomatic initial COVID-19 infection is associated with an increased likelihood of experiencing COVID-19 sequelae – ‘long COVID’ [104], although further studies are needed [105, 106]. ICS may benefit patients with early symptoms by acting on the inflammation evident at the start of infection and by inhibiting viral replication. Managing infection as early as possible has the potential to reduce the incidence of these sequelae, as both the STOIC [17] and PRINCIPLE [18] studies have shown lower symptom persistence in volunteers taking budesonide compared with the standard of care, although both studies were of short (28 days) duration. From the observations of the positive effects of ICS in COVID-19 studies to date, it is possible that they could have a beneficial effect on long-term respiratory outcomes, such as on the impaired lung function associated with COVID-19 sequelae, which warrants investigation.

Additional studies are necessary to determine which of the available ICS compounds may be more likely to reduce the severity of COVID-19, since it is acknowledged that ICS may not all have beneficial effects. For example, there is evidence from a sensitivity analysis of ICS subtypes in a retrospective study of hospitalised patients that budesonide may be associated with a reduced risk of severe COVID-19 outcomes relative to fluticasone, compared with no ICS use [69] and an *in vitro* study demonstrated that ciclesonide and mometasone suppressed SARS-CoV-2 replication, whereas fluticasone did not [91].

Ongoing review of the outcomes of ICS use in patients with COVID-19 is vital to understand if the effect of ICS varies between different strains of SARS-Cov-2 or differs in vaccinated *versus* unvaccinated individuals. What we learn from the effect of ICS on COVID-19 severity will also be crucial in preparation for future pandemics, and the lessons learned could be extended to other viral upper respiratory tract infections in asthma and COPD. While it has been recommended that patients who use ICS should continue to do so [55, 56], it is also important to further investigate ICS use to provide clinicians with clear evidence-based information to relay to patients with chronic lung disease who are concerned about potential increased risk to their health during the pandemic [107]. Further studies investigating the efficacy of ICS in the prevention and treatment of severe disease are also needed. Of note, adverse events such as the onset of diabetic ketoacidosis have been reported in patients with type 2 diabetes mellitus following the use of dexamethasone to treat COVID-19 [108]. As diabetes is one of the most prevalent comorbidities in COVID-19 patients, the early use of ICS to limit infection and reduce the requirement for systemic corticosteroids could reduce the risk of such adverse events in many patients with COVID-19.

To conclude, ICS could potentially benefit COVID-19 infected patients, particularly high-risk groups, if used ideally within days of symptoms starting. As the side effects of ICS are well known and reversible upon cessation, their favourable benefit-to-risk profile would suggest that ICS are a worthwhile consideration for treatment of COVID-19.
